# Micro-evolutionary response of spring migration timing in a wild seabird

**DOI:** 10.1093/evlett/qrad014

**Published:** 2023-05-03

**Authors:** Maria Moiron, Céline Teplitsky, Birgen Haest, Anne Charmantier, Sandra Bouwhuis

**Affiliations:** Life-history Biology Department, Institute of Avian Research, Wilhelmshaven, Germany; CEFE, Univ Montpellier, CNRS, EPHE, IRD, Montpellier, France; CEFE, Univ Montpellier, CNRS, EPHE, IRD, Montpellier, France; Department of Bird Migration, Swiss Ornithological Institute, Sempach, Switzerland; CEFE, Univ Montpellier, CNRS, EPHE, IRD, Montpellier, France; Life-history Biology Department, Institute of Avian Research, Wilhelmshaven, Germany

**Keywords:** adaptation, Breeder’s equation, climate change, common tern, phenology, Robertson’s Secondary Theorem of Selection

## Abstract

In the context of rapid climate change, phenological advance is a key adaptation for which evidence is accumulating across taxa. Among vertebrates, phenotypic plasticity is known to underlie most of this phenological change, while evidence for micro-evolution is very limited and challenging to obtain. In this study, we quantified phenotypic and genetic trends in timing of spring migration using 8,032 dates of arrival at the breeding grounds obtained from observations on 1,715 individual common terns (*Sterna hirundo*) monitored across 27 years, and tested whether these trends were consistent with predictions of a micro-evolutionary response to selection. We observed a strong phenotypic advance of 9.3 days in arrival date, of which c. 5% was accounted for by an advance in breeding values. The Breeder’s equation and Robertson’s Secondary Theorem of Selection predicted qualitatively similar evolutionary responses to selection, and these theoretical predictions were largely consistent with our estimated genetic pattern. Overall, our study provides rare evidence for micro-evolution underlying (part of) an adaptive response to climate change in the wild, and illustrates how a combination of adaptive micro-evolution and phenotypic plasticity facilitated a shift towards earlier spring migration in this free-living population of common terns.

## Introduction

Phenotypic changes in response to climate change are commonplace across taxa (Bellard et al., 2012; Chen et al., 2011; Hendry et al., 2008; Parmesan, 2006; Parmesan & Yohe, 2003; Radchuk et al., 2019), but the mechanism(s) underlying these changes remain largely unknown (Gienapp & Brommer, 2014; Hoffmann & Sgrò, 2011; Lavergne et al., 2010; Merilä & Hendry, 2014; Salamin et al., 2010). Where the mechanisms underlying the observed shifts have been explicitly investigated, in vertebrates they have often been attributed to phenotypic plasticity (i.e., genotypes express variable phenotypes under different environmental conditions), while evidence for adaptive micro-evolution (i.e., genotypes that have a higher fitness increase in frequency in the population) is still relatively rare (Gienapp & Brommer, 2014; Gienapp et al., 2008; Hairston et al., 2005; Henderson, 1963; Hoffmann & Sgrò, 2011; Merilä & Hendry, 2014; Vedder et al., 2013; Visser, 2008).

Animal models (sensu Kruuk, 2004) are useful statistical tools to test for genetic trends and rates of micro-evolutionary responses to selection by means of providing estimates of individual breeding values (i.e., the expected effect of the genes that an individual passes on to its offspring, Falconer & Mackay, 1996; Lynch & Walsh, 1998). The strength of this test, however, relies on properly accounting for uncertainty in breeding value predictions and environmental factors (Hadfield et al., 2010; Postma, 2006). To our knowledge, there are only three long-term studies from natural vertebrate populations that have found evidence for a genetic change underlying a phenotypic change (most likely) in response to climate change, while taking into account the uncertainty in the breeding value estimation and controlling for a temporal trend in the phenotypic data (but see Merilä et al., 2001 and Walsh & Lynch, 2018 for reviews on evolutionary stasis, where no micro-evolutionary change was observed). First, based on 10 years of data on an alpine population of snow voles (*Chionomys nivalis*), Bonnet et al. (2017) showed a genetic change towards a reduced body mass as an adaptive response to viability selection, likely in response to a change in snowfall patterns. This genetic change represented a case of “cryptic evolution” where the population did evolve but there was no observed phenotypic change in body mass. Second, Evans & Gustafsson (2017) studied male plumage coloration in collared flycatchers (*Ficedula albicollis*) over 34 years and showed a marked phenotypic decline, which was mirrored by a decline in the mean breeding value of these males. This decline, which accounted for 11% of the total phenotypic change, was likely driven by viability selection acting on ornamentation being sensitive to the climatic conditions experienced at the breeding ground in the preceding year. And third, using data obtained from a wild population of red deer (*Cervus elaphus*), Bonnet et al. (2019) found that average parturition date had advanced over the 45-year study period, with warmer temperatures during the previous rut season associated with earlier parturition dates, and showed that a micro-evolutionary response accounted for 15% of the total advance of 12.3 days (Bonnet et al., 2019).

Once genetic trends have been estimated, testing their consistency with predictions of a micro-evolutionary response to selection can inform us about the occurrence and magnitude of adaptive responses to selection, as well as our ability to model these responses. Traditionally, the per-generation evolutionary response to selection in a given trait, *R*, can be predicted by *R* = *h*^2^*S*, also known as the Breeder’s equation, where *h*^2^ represents the trait heritability, and *S* is the directional selection differential (Falconer & Mackay, 1996; Lynch & Walsh, 1998). This Breeder’s equation, however, tends to lead to biased predictions in studies from natural populations due to the occurrence of correlated selection as well as confounding environmental effects (i.e., when selection is acting on the environmental rather than genetic component of a trait, the Breeder’s equation will overestimate the trait’s response to selection) (Morrissey et al., 2010). It therefore has been argued that micro-evolutionary responses to selection in natural populations are better predicted by applying Robertson’s Secondary Theorem of natural Selection (hereafter, STS) (Morrissey et al., 2010), which postulates that the additive genetic covariance between a trait and relative fitness is a direct measure of the expected per generation evolutionary change in the mean trait value, *R* (Price, 1970; Robertson, 1966). While STS is a strictly genetic approach to predicting a response to selection, and therefore unbiased by unmeasured covariates, it is also agnostic regarding the selection processes underlying the micro-evolutionary responses. Hence, a comprehensive understanding of the predicted evolutionary responses to selection will benefit from evaluating the predictions from both the Breeder’s equation and the Robertson’s Secondary Theorem of Selection, as they provide complementary information.

Along with poleward shifts in geographic distributions, phenotypic changes in response to global warming are most apparent in phenology (Menzel et al., 2006; Parmesan, 2006; Poloczanska et al., 2013; Root et al., 2003; Thackeray et al., 2010). As such, timing of migration, together with timing of breeding, is a trait of special interest in the context of current global and climate change (Charmantier & Gienapp, 2014; Gienapp et al., 2014; Parmesan & Yohe, 2003). Among birds, most studied populations have shifted their phenology to earlier migration and breeding (review: Charmantier & Gienapp, 2014; Gienapp et al., 2007; Gordo, 2007; Lehikoinen et al., 2004; meta-analysis: Bailey et al., 2022; Radchuk et al., 2019), although not all bird species share the same adaptive potential (e.g., Keogan et al., 2018). For instance, long-distance migrant birds might be constrained in their potential to keep track of changing environmental conditions by inherited circannual clocks and migratory behaviors (Åkesson et al., 2017) and by reduced correlation between climate at the wintering and the breeding grounds, making them more sensitive to rapid warming conditions. With respect to the timing of migration, the date of arrival at the breeding grounds has received most empirical attention, and early arrival is expected to contribute to securing high-quality breeding sites (Kokko et al., 2004) and mates (Coppack & Both, 2002; Ludwigs & Becker, 2005), and to favor an early initiation of breeding (Moiron et al., 2020). Besides often being under strong directional selection (for being early), arrival date has also been found to harbor an important genetic component. For instance, arrival date heritabilities ranged from 0.11 to 0.32 among barn swallows (*Hirundo rustica*, Teplitsky et al., 2011), common terns (*Sterna hirundo*, Arnaud et al., 2013), and great reed warblers (*Acrocephalus arundinaceus*, Tarka et al., 2015), with collective evidence across four bird species revealing a mean heritability of 0.43 (Pulido, 2007).

Even though the prerequisites for a micro-evolutionary response are often met (non-zero additive genetic variance and selection), to date we know of only two studies providing “indirect” evidence for a micro-evolutionary response of the timing of migration in natural bird populations. First, Teplitsky et al. (2011) used two long-term datasets on barn swallows from Spain and Denmark to show that, for the Spanish but not the Danish population, the Breeder’s equation and Robertson’s Secondary Theorem of Selection predicted qualitatively similar responses to selection (towards earlier arrival). It was, however, not estimated whether the study population had undergone substantial phenotypic or genetic change across years, precluding a comparison between observed and predicted responses to selection. Second, Helm et al. (2019) replicated an experimental study of the annual cycle of a long-distance migratory species, the pied flycatcher (*Ficedula hypoleuca*), after 21 years of warming and showed that migration timing had advanced by 9 days. This advance was also observed in a nearby natural population of flycatchers, such that, altogether, their results supported a role of micro-evolution in the earlier spring migration timing.

In our study, we applied a bivariate animal model to test for a micro-evolutionary response in the timing of spring arrival in a long-distance migratory bird. We used 27 years of data from a free-living, pedigreed population of common terns (*Sterna hirundo*). We had two main objectives: (a) to quantify whether there was a detectable genetic change in arrival date (i.e., a temporal trend in individual breeding values) and (b) to test whether this trend was consistent with predictions of micro-evolutionary responses to selection based on both the Breeder’s equation and Robertson’s Secondary Theorem of natural Selection. Previous studies from the same study population found that arrival date was heritable (Arnaud et al., 2013; Moiron et al., 2020) and under directional selection, with earlier arrival being associated with improved reproductive success (Arnaud et al., 2013, but see Ezard et al., 2007) and a higher probability of survival (Zhang et al., 2015b). Hence, although we acknowledge that evolutionary stasis often occurs, even when the prerequisites for micro-evolution are met (Merilä et al., 2001; Walsh & Lynch, 2018), we expected a micro-evolutionary response towards earlier arrival.

## Methods

### Study system and data collection

The data were collected as part of a long-term study of a common tern population located at the Banter See on the German North Sea coast (53° 30ʹ 40″ N, 08° 06ʹ 20″ E). The colony consists of six concrete islands, each surrounded by a 60-cm wall. An individual-based study was initiated in 1992, when 101 adult birds were caught and marked with individually numbered subcutaneously injected transponders. Since 1992, all locally hatched birds have similarly been marked with a transponder shortly prior to fledging and the presence and reproductive performance of marked individuals has been monitored with the help of antennae and following a standard protocol (Becker & Wendeln, 1997). Thanks to the automatic antenna system, affixed to the walls of the colony site since 1994, it is possible to record the arrival date from the wintering grounds of every marked bird in the population. From this, and as confirmed by a tracking study, it is known that the common terns of this population show highly repeatable migratory behavior (Kürten et al., 2022).

As part of the standard protocol, breeding birds are identified using portable antennae placed around each nest for 1–2 days during incubation, which is shared by both partners. Pairs can rear up to three chicks per brood and produce replacement clutches after loss of eggs or chicks. True second clutches (i.e., non-replacement clutches) are extremely rare (1.4% across the study period). Chicks are ringed at hatching and checked every 2–3 days throughout the breeding season until they fledge (at about 26 days; Becker & Wink, 2003) or perish.

### Data selection

The phenotypic and fitness data used in this study were collected between 1994 and 2020. We focused on arrival date at the breeding area from the wintering grounds in West Africa (Becker et al., 2016; Kürten et al., 2022), a phenological trait that captures variation in the timing of spring migration and that is defined as the day of first return to the breeding grounds (1 January = 1) (Zhang et al., 2015a). Because no breeding can occur before a bird has arrived at the colony and recovered from migration, we removed observations of arrival date obtained from the antenna system that were not at least 10 days earlier than the egg laying date (*n* = 696 observations from 390 birds) as they are deemed faulty (Zhang et al., 2015a). The resulting dataset of arrival date included 8,032 observations from 1,715 individuals of known sex and age (mean number of observations per individual = 4.68, range = 1–21). The overall mean (±SD) arrival date across the 27 years was 118 ± 12.63 (~28 April).

We used adult lifetime reproductive success (LRS) as our fitness measure, and quantified it as the total number of fledglings locally produced in the lifetime of an adult individual (Supplementary Figure S1). Although we cannot directly observe an individual’s death, we can reliably assume it, because breeders at the Banter See are highly site-faithful, as evidenced by the resighting probability of breeding individuals being close to one (Szostek & Becker, 2012), and 96% of breeders not skipping recording by the antenna system for two or more consecutive years after first reproduction (Bouwhuis et al., 2015; Zhang et al., 2015a). In addition to using LRS data from individuals assumed to be dead (i.e., not observed in 2019 and 2020; *n* = 946), we also included LRS data from those individuals that were still alive (i.e., observed in 2019 and/or 2020) but older than 10 years (*n* = 121) to avoid “cohort truncation” (i.e., to avoid excluding individuals “not at random” with respect to fitness, Hadfield, 2008; Morrissey et al., 2012a). We chose this threshold because the cumulative reproductive success of known dead birds at ages older than 10 and their lifetime reproductive success are known to be highly correlated (*r* > 0.8, Moiron et al., 2022). This age threshold also matches the biology of the population, as the mean lifespan is ~10 years (Szostek & Becker, 2012). LRS was, however, assigned as “missing” for birds that were observed in 2019 and/or 2020 and younger than 10 years (*n* = 648). As such, the dataset of adult LRS consisted of 1,067 observations from individuals of known age and sex. Details of the social pedigree used in the study can be found in the Supplementary Material.

### Statistical analyses

To test for a trend in phenology across the study period, we ran a linear model with the annual mean arrival dates observed between 1994 and 2020 as a response variable, year as a continuous fixed effect, and assuming a Gaussian error distribution.

To test for a micro-evolutionary change in arrival date, we fitted a bivariate animal model with arrival date and LRS as response variables. Arrival date was modeled assuming a Gaussian error distribution, while LRS was modeled assuming an overdispersed Poisson error distribution with log-link function (Supplementary Figure S1). Because our fitness measure follows a log-normal distribution, variance estimates for absolute fitness on the latent scale data are equivalent to variance estimates directly on the data scale for relative fitness (Bonnet et al., 2019; Morrissey & Bonnet, 2019; de Villemereuil et al., 2016).

The bivariate animal model included random intercepts for individual identity linked to the relatedness matrix (V_A_), for maternal environmental effects (V_MOTHER_; although maternal identity is only known for 38.78% of the 1,715 individuals, see Supplementary Material), and for among-cohort variation (V_COHORT_; note that year of hatching is known for all individuals in this dataset). We also fitted individual identity not linked to the pedigree to account for repeated measures and estimate the permanent environmental effect (V_PE_), and year of breeding (V_YEAR_) as random effects associated only with arrival date. Because, unlike arrival date, LRS has a single measure per individual, it is not possible to fit a covariance at all levels of variation (see Bonnet et al., 2019; Morrissey et al., 2012a). As such, we only modeled the additive genetic covariance between LRS and arrival date (COV_A_), the maternal environmental covariance between LRS and arrival date (COV_MOTHER_), the cohort covariance between LRS and arrival date (COV_COHORT_), and the covariance between the permanent environmental effects on arrival date and the residuals of LRS (as both represent covariance among individuals).

We fitted sex (categorical variable) as a fixed effect associated with both LRS and arrival date. As fixed effects associated only with arrival date, we fitted the linear and quadratic effects of age (continuous variable measured in years, Ezard et al., 2007; Zhang et al., 2015a), the linear effect of year of breeding (continuous variable, to control for the linear temporal trend in phenology), and sea surface temperature at the main wintering area (Kürten et al., 2022) (continuous variable) both as a main effect and in interaction with the linear and quadratic effects of age. We used data of sea surface temperature between 23 June and 27 July the year prior to arrival at the coast of Guinea and Sierra Leone, determined based on the approach developed by Haest et al. (2018, 2019, 2020). All continuous fixed effects (except age) were mean centered and variance standardized.

We estimated the heritability (*h*^2^) of arrival date conditional to the variance explained by fixed effects as the proportion of the total phenotypic variance in arrival date explained by the additive genetic variance.

#### Estimating micro-evolutionary change and genetic drift

From the bivariate animal model described above, we extracted the best linear unbiased predictors (BLUPs) of breeding values of arrival date for each individual and tested for a temporal change. Temporal trends were assessed in two ways, by either regressing the breeding values of arrival date over an individual’s mean breeding year or cohort (i.e., the year of hatching). We used recruited individual data so that results are comparable across the two analyses.

To calculate the probability that the observed change in breeding values differed from a scenario resulting from genetic drift, we simulated random breeding values for arrival date as per Hadfield et al. (2010), and fitted a linear regression to these random breeding values to obtain the temporal slopes due to drift for each posterior sample (temporal slopes were estimated both using an individual’s mean breeding year and cohort). Because the distribution of temporal slopes due to random drift is expected to be zero-centered (sampling was neutral), we could then calculate the proportion of the posterior distribution of these drift slopes that returned a regression value more negative than the posterior mode of the observed temporal slopes.

Finally, the annual estimate of a micro-evolutionary response estimated as the change in breeding values was converted to a per-generation rate by multiplying it by the population’s generation time of ~7.95 years. We estimated this generation time for common terns in our dataset as the mean age of parents of nestlings that later recruit in the population (Charlesworth, 1994), 8 years being in line with the generation time previously estimated in this species (Nisbet et al., 2020). We also converted the annual estimate of micro-evolution to units of standard deviation per generation by multiplying by generation time and dividing by phenotypic standard deviations. This way, the unit for evolutionary change was equivalent to a change measured in Haldanes (Hendry & Kinnison, 1999).

#### Predicting micro-evolutionary change

We predicted the expected per‐generation rate of micro-evolutionary change of arrival date by first applying the Breeder’s Equation (i.e., *R* = *h*^*2*^*S*). The selection differential or phenotypic covariance between focal trait and fitness, *S*, was calculated as the sum of the additive genetic and permanent environmental covariances between arrival date and adult LRS. To obtain the full posterior distribution for the evolutionary response to selection (*R*) and hence account for the uncertainty in all estimated parameters, we multiplied the full posterior distributions of *h*^2^ and *S*. Second, Robertson’s Secondary Theorem of natural Selection states that the additive genetic covariance between a trait and relative fitness represents a direct measure of the expected per‐generation evolutionary change *R* in that trait (Price, 1970; Robertson, 1966). Both estimates of evolutionary response to selection (*R*) were reported as the posterior mode and 95% credible intervals.

#### Statistical model implementation

We fitted the model using a Bayesian framework implemented in the statistical software R (v. 4.0.5, R Core Team, 2019) using the R-package *MCMCglmm* (Hadfield, 2010). We fitted a parameter‐expanded prior. The number of iterations and thinning interval were chosen to ensure that the minimum MCMC effective sample size for all parameters was 1,000. Burn-in was set to a minimum of 2,000 iterations. The retained effective sample size yielded absolute autocorrelation values lower than 0.1 and satisfied convergence criteria based on the Heidelberger and Welch convergence diagnostic (Heidelberger & Welch, 1981). We drew inferences from the posterior mode and 95% Credible Intervals (95% CI).

## Results

### Temporal trend in arrival date

**Figure 1.**
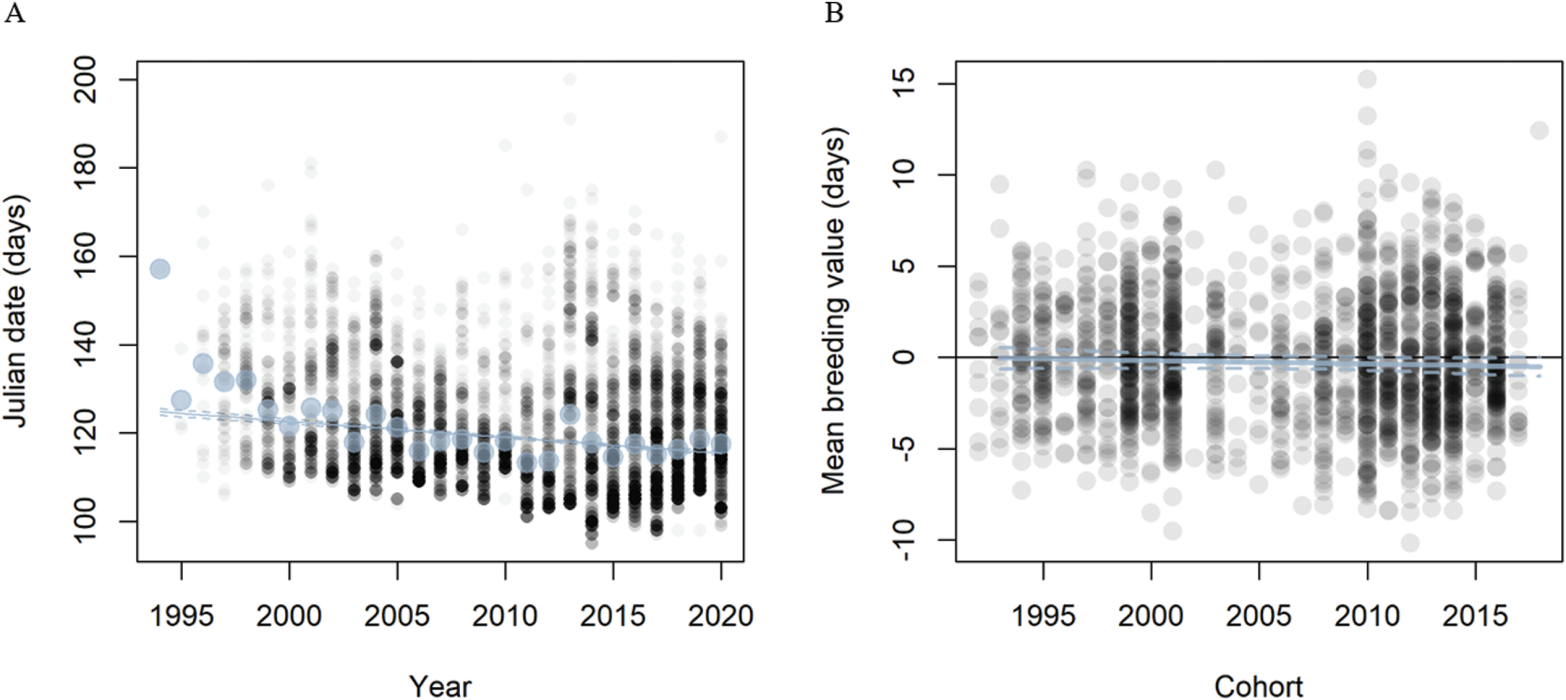
Temporal phenotypic trends in mean arrival date across years (A) and in individual breeding values in arrival date across cohorts (i.e., year of hatching) (B). Breeding values were extracted from the bivariate animal model of arrival date and adult lifetime reproductive success reported in Table 1. In (A), large silver dots represent annual means, small grey dots individual observations, and silver lines the slope and associated 95% confidence interval of the phenotypic trend across the 27 years of study. In (B), grey dots represent individual breeding values of arrival date; black solid line represents no change in breeding values; silver lines are the slope and associated 95% confidence interval of the temporal trend in breeding values.

Between 1994 and 2020, the annual mean arrival date advanced at a rate of 0.36 days per year (overall change: −9.34 days, 95% CI: −10.43, −8.24; Figure 1A), as did the individuals’ mean arrival date regressed over their mean breeding year, with a rate of 0.09 days per year (overall change: −2.45 days, 95% CI: −4.51, −0.38).

**Table 1.** Estimates from a bivariate animal model of arrival date and adult lifetime reproductive success (LRS).

Fixed effects	β (95% CI)	
Intercept_arrival date_	156.356	[154.7, 158.439]
Intercept_fitness_	0.857	[0.573, 1.191]
Age [linear]_arrival date_	−8.4	[−8.655, −8.058]
Age [quadratic]_arrival date_	0.368	[0.35, 0.385]
Sea surface temperature_arrival date_	−1.565	[−2.834, 0.265]
Sea surface temperature × age [linear]_arrival date_	0.158	[−0.055, 0.4]
Sea surface temperature × age [quadratic]_arrival date_	−0.013	[−0.023, −0.001]
Year_arrival date_	−4.302	[−5.669, −2.394]
Year × age [linear]_arrival date_	1.26	[1.071, 1.596]
Year × age [quadratic]_arrival date_	−0.102	[−0.12, −0.092]
Sex [female]_arrival date_	−1.637	[−2.221, −1.012]
Sex [female]_fitness_	0.1	[−0.066, 0.208]

Random effects	σ^2^ (95% CI)	
V_A arrival date_	22.425	[15.343, 29.651]
V_A fitness_	0.002	[0, 0.268]
COV_A arrival date−fitness_	−0.057	[−1.055, 0.271]
V_MOTHER arrival date_	0.011	[0, 2.334]
V_MOTHER fitness_	0.001	[0, 0.123]
COV_MOTHER arrival date−fitness_	0	[−0.176, 0.115]
V_COHORT arrival date_	0.813	[0, 2.186]
V_COHORT fitness_	0.389	[0.166, 0.843]
COV_COHORT arrival date−fitness_	−0.181	[−0.825, 0.178]
V_PE arrival date_	8.807	[1.419, 12.828]
V_YEAR arrival date_	7.135	[4.01, 14.715]
V_R arrival date_	52.614	[50.918, 54.462]
V_R fitness_	0.809	[0.621, 1.009]
COV_PE arrival date – R fitness_	−0.636	[−1.38, −0.047]

*Note.* Estimates shown represent V_A_ for additive genetic variance, COV_A_ for additive genetic covariance, V_PE_ for permanent environmental variance, (CO)V_MOTHER_ for maternal environmental (co)variances, (CO)V_COHORT_ for cohort (co)variances, V_YEAR_ for among-year variance, and V_R_ for residual variance. All continuous fixed effects, except age, were mean centered and variance standardized. Estimates represent posterior modes with associated 95% credible intervals.

### Sources of phenotypic variation in arrival date and adult LRS

The mean population age increased from 2 to 8 years over the first 15 years of the study, before leveling off (Supplementary Figure S2). Arrival date was influenced by the linear and quadratic effects of age, as well as by sea surface temperature at the main wintering area and year of breeding in interaction with age. On average, birds arrived earlier following years of warmer temperatures at the wintering area and in the later years of the study period, although these effects depended on the age of the individuals, with mature adult birds arriving earlier and showing a weaker age-dependent decline than younger birds (Table 1). Arrival date was also influenced by the sex of individuals: females arrived earlier than males (Table 1).

Interpreting the variance components, common terns showed strong heritable differences in arrival date: additive genetic effects accounted for 25.74% of the total phenotypic variance, while permanent environmental effects accounted for an additional 7.17% (Table 1). Arrival date also harbored variance among years of breeding (0.84%, Table 1). Maternal environmental and cohort effects were close to, or not significantly different from, zero (Table 1), although we may have limited statistical power to estimate the maternal environmental effect. Additionally, we found the additive genetic variance in adult LRS to be close to, or not significantly different from, zero (Table 1). While this is a pattern commonly observed in fitness and fitness components (reviewed by Hendry et al., 2018), the close-to-zero estimate might be due to either a true absence of additive genetic variance in fitness or a lack of power to detect it with higher precision. The latter case is true for several fitness components in this population (Moiron et al., 2022).

### Estimating micro-evolutionary change and genetic drift

The best linear unbiased predictors (BLUPs) for breeding values of arrival date extracted from the bivariate animal model described above were tested for a trend over time, that is, over an individual’s mean breeding year or across cohorts. Using cohort for the temporal trend, the slope of the linear regression was estimated at −0.021 days per year of breeding (95% CI: −0.056, 0.016), indicating that breeding values advanced ~0.4 days across the 27-year study (Figure 1B). While the lower 95% CI of the annual estimate was overlapping zero, the fraction of the posterior distribution of the genetic slopes that was greater than zero was only 14%. Re‐expressed in units of phenotypic standard deviations per generation, the estimated rate of micro-evolution corresponded to an observed evolutionary change of −0.003 Haldanes (95% CI: −0.012, 0.003), which can also be expressed as −0.129 days per generation (95% CI: −0.449, 0.126). Using an individual’s mean breeding year for the temporal trend, we observed a similar pattern: the slope of the linear regression was estimated at −0.021 days per year (95% CI: −0.074, 0.018, Supplementary Figure S4). Finally, less than 8.3% of the simulations of random drift generated an advance as large as, or larger than, the change estimated from the linear regression of genetic change using either cohort or an individual’s mean breeding years (Supplementary Figure S3).

### Predicting micro-evolutionary change

The phenotypic selection differential for arrival date quantified as the sum of additive genetic and permanent environmental covariances between the trait and fitness (Table 1) was negative and the associated 95% credible intervals did not overlap zero (*S* = −1.058, 95% CI: −1.609, −0.592), indicating that individuals with earlier arrival dates obtained, on average, higher adult LRS.

**Figure 2.**
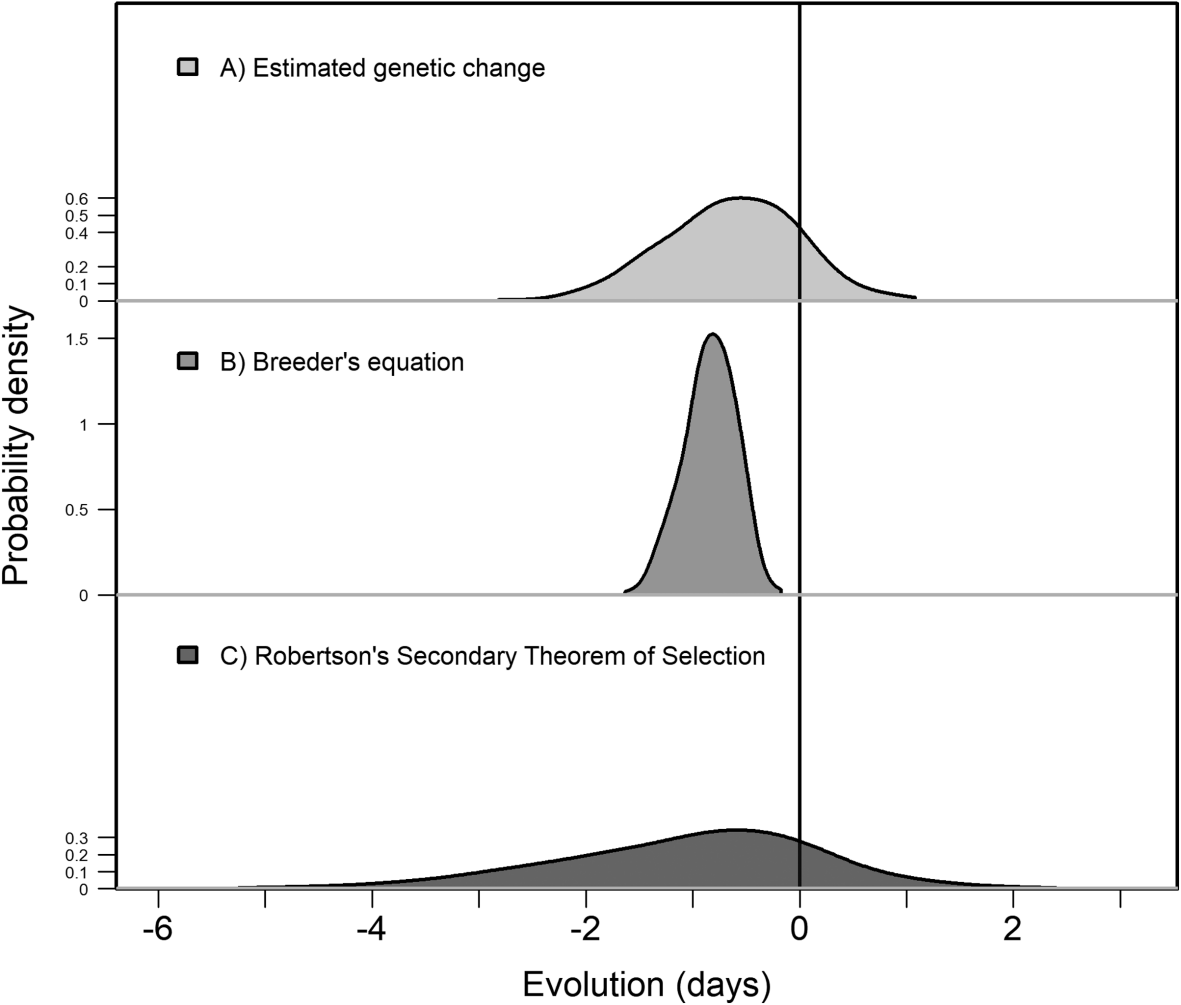
Posterior distributions for the estimated and predicted evolutionary response of arrival date over the 27-year study period. From top to bottom, (A) “Estimated genetic change” was measured as the temporal change in individual breeding values for arrival date, (B) the predicted rate of evolutionary response based on the “Breeder’s equation” was quantified using a bivariate animal model from which we extracted the estimates of selection differential (individual-level covariance between arrival date and adult lifetime reproductive success) and heritability of arrival date, and (C) the predicted rate of evolutionary response based on the “Robertson’s Secondary Theorem of Selection” was quantified as the additive genetic covariance between arrival date and adult lifetime reproductive success. All estimates presented were converted to an evolutionary change (“evolution”) measured in days of change over the 27-year study period. Parameter estimates are summarized in the main text. All distributions have the same area and axes scales.

Given that arrival date was heritable and under directional selection (Table 1), the Breeder’s equation predicted a rate of evolutionary change in arrival date of −0.029 days per year (95% CI: −0.050, −0.015), equivalent to a total change of −0.742 days across the 27 years of study (95% CI: −1.297, −0.432, Figure 2), and translating into a response rate of −0.228 days per generation (95% CI: −0.339, −0.119) and −0.018 Haldanes (95% CI: −0.032, −0.009).

The bivariate animal model of adult LRS and arrival date revealed a negative additive genetic covariance between arrival date and LRS (COV_A_ = −0.057, 95% CI: −1.055, 0.271, Table 1), corresponding to a total evolutionary change in arrival date of −0.185 days across the 27-year study period (95% CI: −3.430, 0.880; Figure 2), or a predicted evolutionary rate of −0.007 days per year (95% CI: −0.132, 0.034) and −0.001 Haldanes (95% CI: −0.084, 0.021). While the point estimate for the Robertson’s Secondary Theorem of natural Selection also predicted an advance in arrival date, the 95% CI of the predicted evolutionary response was wide and the lower 95% CI limit overlapped with zero (Table 1). Nevertheless, 82.2% of the posterior samples were below zero, suggesting the additive genetic covariance between arrival date and LRS to be very likely negative, but we might lack the statistical power to detect it with more certainty.

## Discussion

In this study, we investigated a case of expected evolutionary change in the timing of spring migration of a seabird to further our understanding of the evolutionary dynamics of phenology in natural populations, whilst also unraveling the selection pressures that underlie micro-evolutionary changes. We did so by using long-term phenotypic data from a free-living population of common terns located at the North Sea coast of Germany and applying a bivariate animal model that allowed incorporating relatedness information from a social pedigree.

Previous studies from the common tern population at the Banter See found that the timing of migration from the wintering grounds was heritable (*h*^2^ = 0.06 ± 0.03, Arnaud et al., 2013), and under directional selection (Arnaud et al., 2013, but Ezard et al., 2007). In the current study, we corroborated those earlier findings using a considerably longer and larger dataset, finding a heritability of 24.7%, and a phenotypic selection differential of −1.1 days. Given that the prerequisites for a potential evolutionary change were met, we expected an evolutionary response to selection with advanced arrival dates from the wintering grounds, that is, a change in mean phenotype towards earlier arrival dates at the genetic level, although apparent evolutionary stasis is widespread (see Table 1 in Merilä et al., 2001 and Table 20.3 in Walsh & Lynch, 2018), and phenotypic plasticity and/or other sources of non-genetic variation might also lead to phenotypic divergence (e.g., Bonnet et al., 2017, 2019; Gienapp & Merilä, 2014; Gienapp et al., 2008). Indeed, a previous study from this population of common terns found support for individual, and, to a lesser extent, additive genetic variance in the plasticity of arrival date in response to an important climatic factor at the main wintering areas (Moiron et al. *in revision*), although that was not the case for the plasticity of timing of breeding (Dobson et al., 2017).

We found the average arrival date to have advanced 9.34 days across the 1994–2020 study period (95% CI ranged from −10.43 to −8.24, Figure 1A). Likewise, there was a temporal trend in the average breeding value for arrival date (Figure 1B). Taking into account the parameters’ uncertainties and early criticisms on the use of predicted breeding values (Hadfield et al., 2010; Postma, 2006), we found that the expected response to selection ranged from −0.056 to 0.016 days per year when using cohort to assess the temporal trend. Whereas the 95% credible intervals of the observed genetic change overlapped with zero, the posterior probability of this change being greater than zero was only 14% (Figure 2). Additionally, the probability of random genetic drift as the sole driver of the evolutionary change to generate a trend in breeding values as strong as, or stronger than, the observed genetic trend was only 8.3%, indicating that an evolutionary response to selection is a much more likely explanation for the observed temporal change in arrival date than genetic drift.

### Theoretical predictions of evolutionary change

We estimated all parameters associated with the Breeder’s equation and Robertson’s Secondary Theorem of Selection (STS), for example, heritability, selection differential, and genetic covariance between the focal phenotype and relative fitness, using a single Bayesian animal model. This approach allows for a quantitative comparison of both evolutionary change predictions, while also taking forward the uncertainties in the estimated parameters and subsequent calculations. The predicted rate of evolutionary change in arrival date based on the Breeder’s equation was −0.018 Haldanes (95% CI: −0.032, −0.009), indicating that common terns were expected to advance the timing of their spring migration over time. The predicted rate of evolutionary change in arrival date based on the STS was −0.001 Haldanes, similarly indicating a response to selection towards earlier spring migration dates, although the associated 95% credible intervals for this estimate were overlapping zero (95% CI: −0.084, 0.021).

Altogether, the theoretical predictions of evolutionary change based on the Breeder’s equation and Robertson’s Secondary Theorem of Selection were largely concordant, both in direction and strength, although the STS estimate was associated with substantial statistical uncertainty. In addition, these two theoretical predictions of evolutionary change were qualitatively similar to the estimated genetic change (i.e., the posterior distributions for the three parameters largely overlapped, Figure 2), and in line with the observed temporal trend in the mean phenotype (Figure 1A). As such, our results provide a rare case of agreement in theoretical predictions of evolutionary change that are consistent with observed genetic patterns.

Additionally, and given that we found the evolutionary predictions from the Breeder’s equation and STS to be in substantial agreement, our finding implies that the Breeder’s equation might be a good predictor of evolutionary change in our study system, where the assumption of causality most likely holds, at least, when assuming a static environment (Kruuk et al., 2002, 2003; Morrissey et al., 2010, 2012a; Queller, 1992; Rausher, 1992). It also implies that our selection estimate might be mostly unbiased, that is, the individual-level covariance between arrival date and relative fitness is mostly caused by arrival date, and there are no “missing traits” (Hadfield, 2008; Morrissey et al., 2010, 2012b; Queller, 1992). These results shed light on the true form of natural selection acting on spring migration timing and suggest a lack of genetic constraints that might interfere with an evolutionary response to selection. However, it is important to note that, while our three estimates of evolutionary response largely agreed, conclusions must be drawn with care as we cannot readily neglect the potential for the apparent agreement in evolutionary predictions to be coincidental. Multiple factors can potentially bias evolution estimates (e.g., a “missing fraction” in our fitness estimate or indirect selection from genetically-correlated traits), most often affecting evolutionary predictions based on the Breeder’s equation, but also on STS (Kruuk et al., 2002, 2003; Merilä et al., 2001; Morrissey et al., 2012a; Walsh & Lynch, 2018).

### Global warming as underlying driver of phenotypic change

Temperatures in the wintering grounds of the studied population have significantly increased since 1994 (sea surface temperature change in West Africa between 1994 and 2020 = 0.66°C, 95% CI: 0.16, 1.16), and such warming has been associated with earlier spring phenological events (Dobson et al., 2017), making climate warming an obvious mechanism potentially underlying the population’s change in migration timing. However, given the observational nature of our study and the complex life cycle of long-distance migratory species experiencing environmental conditions in both hemispheres, we cannot test this hypothesis directly and identify the true biological cue. We can, however, speculate about the potential benefits of earlier arrival from the wintering grounds. Such early arrival could facilitate access to higher-quality breeding sites (Kokko et al., 2004) and mates (Coppack & Both, 2002; Ludwigs & Becker, 2005), and an early initiation of breeding. Early breeding, in turn, would allow for the production of replacement clutches in cases of predation, accidental egg or chick loss, or poor food availability for rearing offspring early in the season, and such replacement clutches (8.9% of clutches across the study period) indeed are known to significantly contribute to LRS (Becker & Zhang, 2011). As such, selection for earlier timing of breeding may translate into selection for earlier timing of spring migration. Additionally, evolutionary responses in arrival date likely are aided by assortative mating for migration timing (Bearhop et al., 2005; see also Moiron et al., 2020).

### Relative contributions of evolutionary and plastic responses to phenotypic change

The estimated genetic change in the timing of spring migration represented c. 5% of the population’s phenotypic change, indicating that the observed advance in migration timing was mostly underpinned by other sources of environmental (non-genetic) variation, such as changes in the population’s age structure (Supplementary Figure S2) or phenotypic plasticity. Indeed, a recent study identified a plastic response of arrival date to climate change, with birds arriving earlier at the breeding ground following warmer years at the main wintering area (Moiron et al., in revision). Altogether, our findings therefore are fully in line with general reports of phenotypic plasticity underlying the vast majority of phenotypic responses to climate change (Charmantier & Gienapp, 2014; Merilä & Hendry, 2014; Parmesan, 2006 and references therein; Vedder et al., 2013), but also highlight that the existence of phenotypic plasticity does not readily imply a lack of a micro-evolutionary response or preclude their simultaneous existence, even in relatively long-lived species (e.g., Bonnet et al., 2019).

## Concluding remarks

While plastic responses to climate change are widely reported across taxonomic groups, evidence of micro-evolutionary responses to selection is still uncommon, and considerably more challenging to accrue, mostly because the data collection for documenting micro-evolution in the wild remains a long-term task. In this study, we provided rare evidence of an evolutionary change in spring phenology, likely in response to global warming, while also illustrating how both plastic and genetic changes can simultaneously facilitate phenotypic divergence in natural populations. Our work further emphasizes the importance of maintaining long-term individual-based studies of natural populations to unravel the diversity of biological responses to climate change, and, generally, to understand selective patterns and evolutionary dynamics of phenotypic traits in the wild.

## Supplementary material

Supplementary material is available online at *Evolution Letters* (https://academic.oup.com/evlett/qrad014).

qrad014_suppl_Supplementary_Material

## Data Availability

Data has been archived in the Dryad Digital Repository (https://doi.org/10.5061/dryad.8kprr4xqj).
