# Supplementary material for: Micro-evolutionary response of spring migration timing in a wild seabird
Source: Evol Lett. 2023 May 3;8(1):8–17. doi: 10.1093/evlett/qrad014 (PMC10872114; doi:10.1093/evlett/qrad014)

Micro-evolutionary response of spring migration timing in a wild seabird

Maria Moiron, Céline Teplitsky, Birgen Haest, Anne Charmantier, Sandra Bouwhuis

—Online Supplementary Material—

Supplementary Text S1. Pedigree information….....Page 2

Supplementary Figure S1….....Page 3

Supplementary Figure S2….....Page 4

Supplementary Figure S3….....Page 5

Supplementary Figure S4….....Page 6

SUPPLEMENTARY TEXT S1. PEDIGREE

A social pedigree was constructed from the observations of parents and their fledged offspring. Due to the very low levels of extra-pair paternity in the population (97.1% of true siblings found among 22 broods, González-Solís et al. 2001), the social pedigree is a good approximation of the genetic pedigree. For the period 1994-2020, the pruned pedigree comprised 1807 individuals. The maximum depth was five generations, the number of paternities and maternities 671 and 651, respectively. The number of full sibs was 235, that of maternal and paternal sibs 721 and 834, respectively. The histogram below represents the relatedness between pairs of individuals present in the pruned pedigree (black line: number of pairs for each level of relatedness, grey dot: no pair was found at this level).

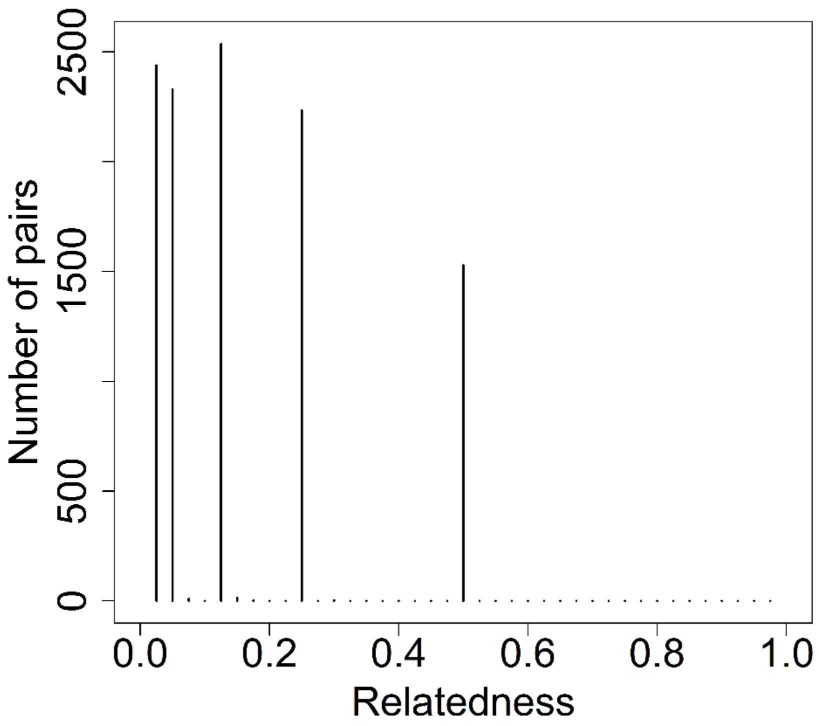

Figure S1. Phenotypic distribution of adult Lifetime Reproductive Success measured as the total number of fledglings locally produced in the lifetime of an adult individual.

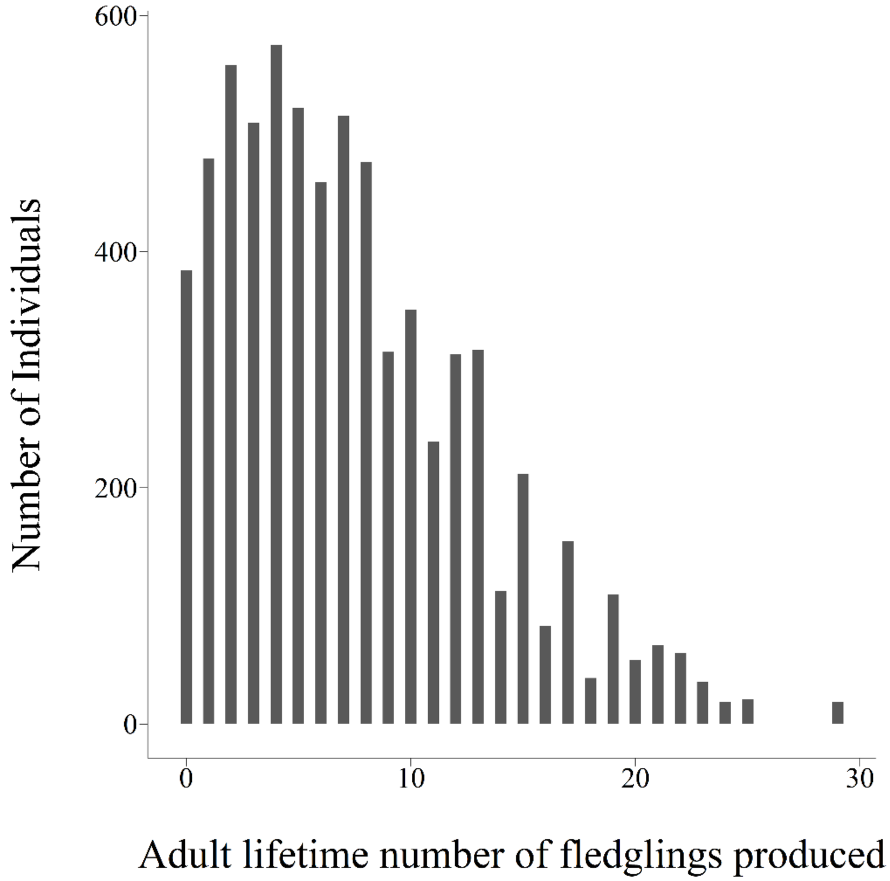

Figure S2. Population change in age over the 27-year study period. Circles represent annual means and bars standard errors.

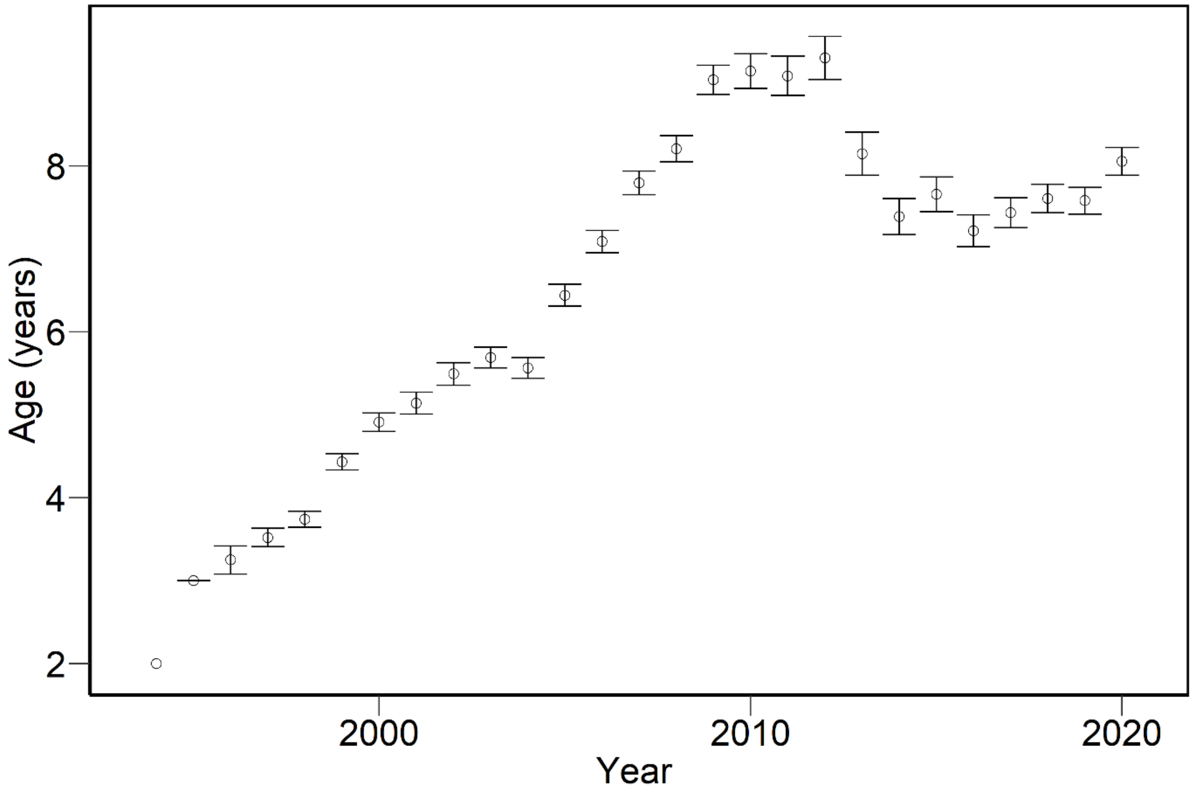

Figure S3. Posterior MCMC samples (bars), kernel density estimation (solid black line), posterior mean (red dotted line), and 95% Credible Intervals (black dashed lines) for simulations of genetic drift as a potential mechanism driving the estimated evolutionary change.

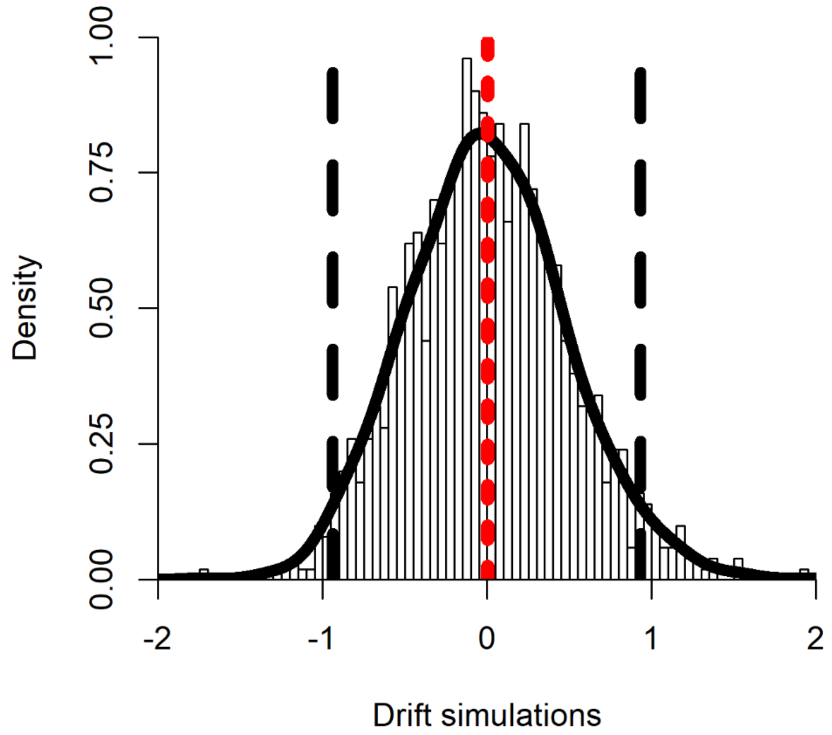

Figure S4. Temporal phenotypic trend in individual breeding values in arrival date over an individual's mean breeding year. Breeding values were extracted from the bivariate animal model of arrival date and adult Lifetime Reproductive Success (Table 1). Grey dots represent individual breeding values of arrival date; silver lines are the slope and associated 95% Confidence Interval of the temporal trend in breeding values.

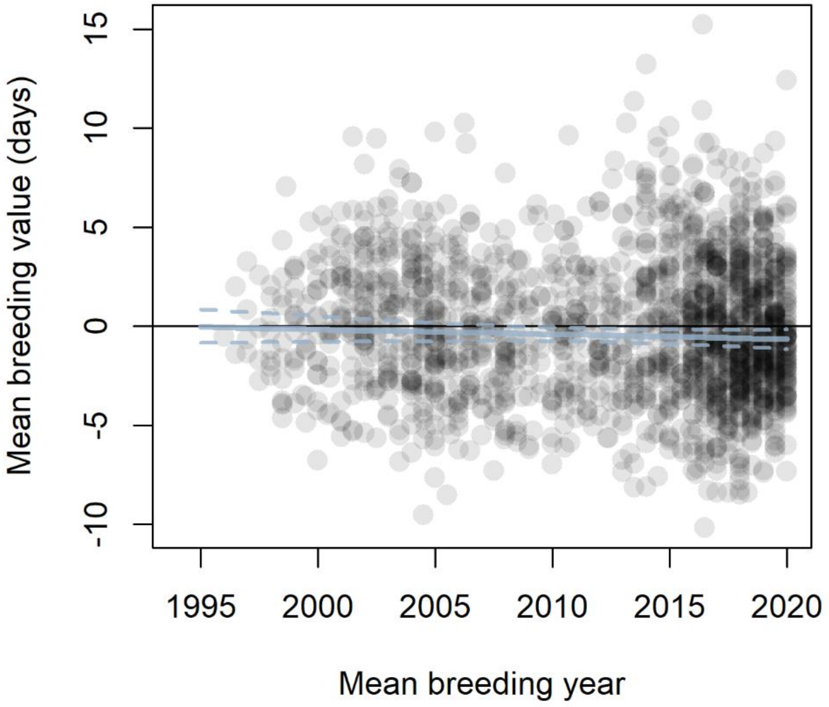

Supplement: qrad014_suppl_Supplementary_Material [file qrad014_suppl_supplementary_material.pdf]
